# Intrinsic and extrinsic drivers of source–sink dynamics

**DOI:** 10.1002/ece3.2029

**Published:** 2016-02-22

**Authors:** Julie A. Heinrichs, Joshua J. Lawler, Nathan H. Schumaker

**Affiliations:** ^1^ School of Environmental and Forest SciencesUniversity of WashingtonP.O. Box 352100SeattleWA98195‐2100; ^2^ Western Ecology DivisionU.S. EPA200 SW 35^th^ St.CorvallisOregon97333

**Keywords:** Habitat quality, individual‐based model, landscape pattern, population growth, source‐sink dynamics, stochasticity

## Abstract

Many factors affect the presence and exchange of individuals among subpopulations and influence not only the emergence, but the strength of ensuing source–sink dynamics within metapopulations. Yet their relative contributions remain largely unexplored. To help identify the characteristics of empirical systems that are likely to exhibit strong versus weak source–sink dynamics and inform their differential management, we compared the relative roles of influential factors in strengthening source–sink dynamics. In a series of controlled experiments within a spatially explicit individual‐based model framework, we varied patch quality, patch size, the dispersion of high‐ and low‐quality patches, population growth rates, dispersal distances, and environmental stochasticity in a factorial design. We then recorded source–sink dynamics that emerged from the simulated habitat and population factors. Long‐term differences in births and deaths were quantified for sources and sinks in each system and used in a statistical model to rank the influences of key factors. Our results suggest that systems with species capable of rapid growth, occupying habitat patches with more disparate qualities, with interspersed higher‐ and lower‐quality habitats, and that experience relatively stable environments (i.e., fewer negative perturbations) are more likely to exhibit strong source–sink dynamics. Strong source–sink dynamics emerged under diverse combinations of factors, suggesting that simple inferences of process from pattern will likely be inadequate to predict and assess the strength of source–sink dynamics. Our results also suggest that it may be more difficult to detect and accurately measure source–sink dynamics in slow‐growing populations, highly variable environments, and where a subtle gradient of habitat quality exists.

## Introduction

Spatial variation in habitat quality is the basic factor that structures source–sink dynamics in heterogeneous landscapes (Pulliam 1988; Dias 1996). Demographic surpluses in higher‐quality habitats (e.g., sources) and deficits in lower‐quality habitats (e.g., sinks) commonly arise, and movement among local populations can stabilize dynamics at regional scales (Dias 1996). At steady state, some local populations become the net exporters of individuals (i.e., sources) where births outweigh deaths (*b* > *d*) and emigrants outnumber immigrants (*e* > *i*), and other populations become net importers (i.e., sinks) where the opposite demographic and movement conditions hold (Pulliam 1988). Although differences in habitat quality (see Hall et al. 1997) provide a basis for source–sink dynamics to emerge, other habitat and population characteristics can affect reproduction and survival through space and time, and play a role in strengthening or weakening source–sink dynamics (Dunning et al. 1992). Habitat characteristics including differences in patch sizes and qualities, as well as the proximity of high‐quality to low‐quality habitats, have the potential to influence the strength of sources and sinks. Similarly, species and population factors such as growth rates, dispersal abilities, and demographic responses to environmental variability can affect the severity of source–sink dynamics, driving sources, and sinks to become more or less extreme. Although these habitat and population factors have been individually found to affect source–sink dynamics, their relative importance is not well understood.

Populations are increasingly conceptualized and managed based on their source–sink status or the suspected presence of source–sink dynamics within the system. Hence, there is a clear practical need to be able to distinguish among source and sink populations. Differential management of sources and sinks can be particularly important in avoiding the counterproductive actions associated with falsely assuming that an animal's realized niche in a sink habitat represents their fundamental niche (Pulliam and Danielson 1991; Boughton 2000). Further, the large continuum of source–sink strengths ranging from minor asymmetries to overwhelming directional flows of individuals suggests that an understanding of the strength of the system and the factors that augment or diminish its strength has the potential to guide effective decisions and actions. Yet to date, studies have primarily evaluated the conditions under which source–sink dynamics are incited, with the limited evaluations of the factors that strengthen dynamics once incited.

Source–sink literature points to dispersal and habitat selection behavior as providing the basis for emergent source–sink dynamics in heterogeneous landscapes, directing the flow of individuals among habitats and resulting birth, death rates, and local densities. Source–sink dynamics can arise as a result of random dispersal (e.g., diffusion), as well as with passive dispersal mechanisms (e.g., exchange of a fixed or stable proportion of dispersers among asymmetrically sized populations; Boughton 2000). Ideal preemptive habitat selection behavior can also lead to source–sink dynamics, with animals that arrive first preempting the use of the best sites, maximizing their reproductive output and fitness. As population density increases, late arrivals are forced to settle in lower‐quality habitats, potentially leading to lower reproductive success, higher mortality, and the creation of population sinks (Pulliam and Danielson 1991). In empirical populations, realistic animal dispersal and habitat selection can incorporate all these elements into complex decision processes that influence ensuing source–sink outcomes. We combine these forces to incite source–sink dynamics in a range of landscapes and life history characteristics, using a novel approach that incorporates mechanistic density‐dependent habitat selection, the concepts of passive diffusion in emigrating from disparate‐sized habitat patches, diffusion in the form of quasirandom walks through matrix to find habitat, and ideal preemption in excluding latecomers from the best sites.

To compare the relative influences of habitat and population factors on the strength of emergent source–sink systems, we simulated metapopulation dynamics in a range of controlled landscapes, using realistic movement and territorial animal behavior within the context of a spatially explicit individual‐based population model. We used a set of hypothetical landscapes and species and six potential drivers (population growth rates, differential patch qualities and sizes, patch quality patterns, dispersal distances, and levels of environmental variation), in a factorial design to rank the influence of the different factors. To assess the consistency of landscape and population drivers of source–sink strength, we also examined their relative influence among alternative habitat selection scenarios with varying degrees of awareness of the landscape, habitat quality, and abilities to optimize fitness. Many animals occupy sink habitats, resulting from an inability to discern adverse fitness consequences (i.e., an ecological trap; Howe et al. 1991; Battin 2004), an unwillingness to emigrate elsewhere (e.g., strong site fidelities), or an improbability of surviving to the next opportunity to relocate (e.g., high overwinter mortality for kangaroo rats; Holt and Gaines 1992; Heinrichs et al. 2010). With limited knowledge of the landscape, we expected animals that are better able to detect habitat quality to select fitness‐optimizing territories to a greater extent than less discerning animals, producing weaker source–sink systems (and the converse to have stronger dynamics).

We expected the strength of source–sink systems to depend on habitat and populations factors, influencing local population densities and density‐dependent emigration, and to be driven by differences in habitat quality. As patch occupancy is a necessary precondition for local habitat quality to be important, factors increasing th size of potential colonizing populations were expected to strengthen source–sink dynamics. The ability for local populations to grow, reach carrying capacities, and induce density‐dependent emigration and fitness consequences was expected to be a key driver of source–sink strength. We also expected that systems that were not challenged by periodic population depressions would produce stronger sources and sinks than those affected by perturbations. We expected that greater accessibility and propensity of individuals in sources to diffuse to sinks would strengthen source–sink dynamics. At stable state, we expected asymmetric patch sizes to strengthen system dynamics through passive dispersal mechanisms. Lastly, Species with longer dispersal distances relative to their territory size were expected to select better territories and maximize their fitness to a greater degree than shorter dispersers, weakening source–sink dynamics. Although we do not directly derive hypotheses for empirical populations, these general expectations lend themselves to thorough testing with empirical data. If supported, these hypotheses could be used to indicate systems with a high degree of correlation and dependency among populations, the degree to which density‐dependent regulation may be influencing population outcomes, and where source–sink dynamics may require additional effort to detect and quantify.

## Methods

### Approach

To explore the influences of alternative landscapes and species and population traits, we designed a series of simple neutral landscapes (Gardner and O'Neill 1991) and ecological profiles (e.g., Vos et al. 2001; Wiegand et al. 2005). The neutral habitat models produced an array of landscapes that varied in patch size disparity, patch quality disparity, and proximity of high‐quality to low‐quality patches, while holding landscape conditions (habitat amount, overall quality, and fragmentation) constant. The ecological profiles were designed to represent a wide range of population growth rates (fecundity), dispersal distances, and responses to environmental variation. A spatially explicit individual‐based model was then used to quantify emergent source–sink dynamics in all combinations of neutral landscapes and ecological profiles. Animal births, movements, and deaths were recorded throughout the landscape and used to assess the long‐term difference in births and deaths for each local population, hereafter referred to as productivity. For each scenario, the difference in productivity among sources and sinks was used to measure source–sink strength. The influence of each landscape and population trait was assessed and ranked using linear regression.

### Landscapes

**Figure 1:**
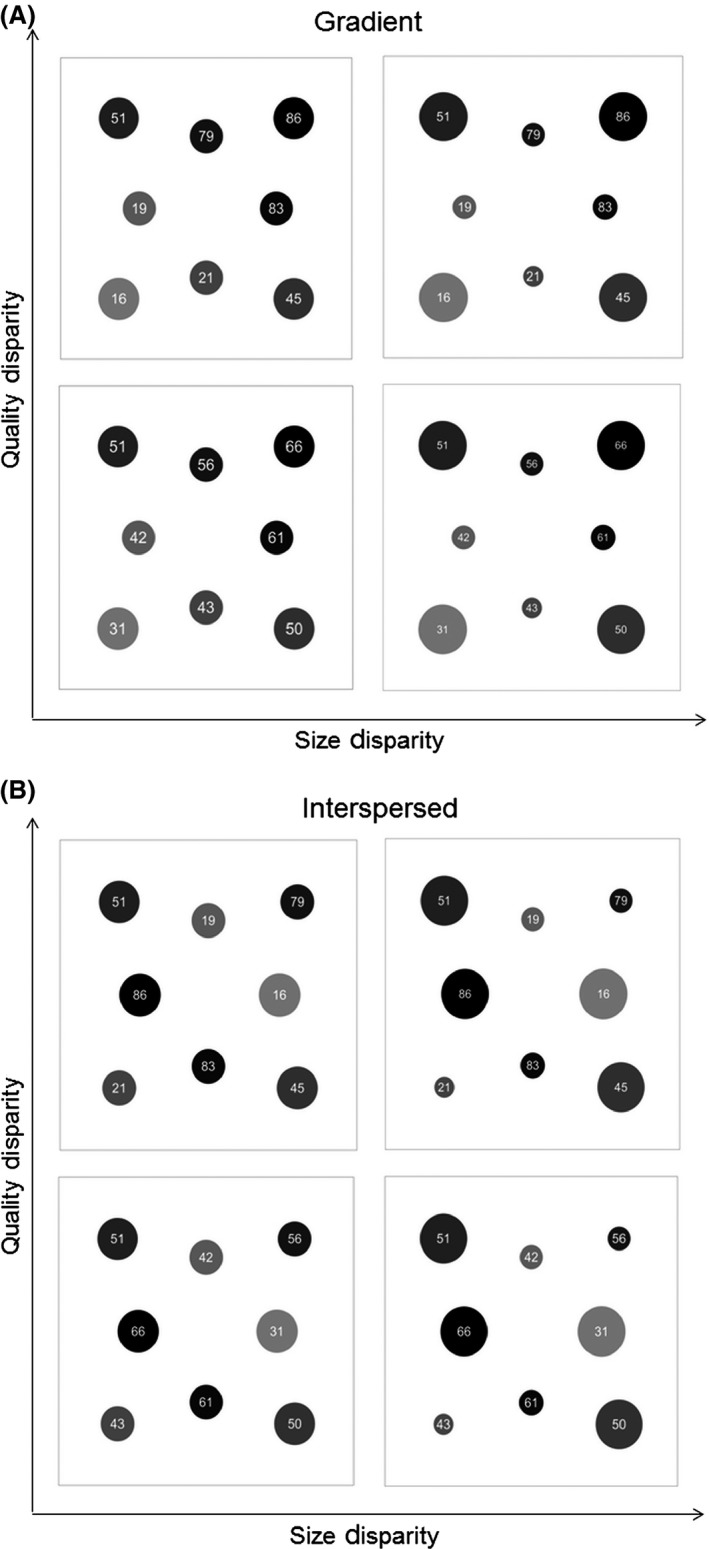
Neutral landscapes varying in patch size disparity (*x* axes), patch quality disparity (*y* axes; with higher numbers representing greater resource scores), and spatial gradient (A) or interspersion (B) of high‐ and low‐quality patches.

We developed eight equivalent landscapes, each consisting of eight discrete circular patches embedded in an uninhabitable matrix which was permeable to movement (Fig. 1). Landscapes varied in the disparity of patch sizes and qualities, as well as the proximity of high‐ to low‐quality patches, according to a factorial design. Although the distribution of patch sizes and qualities varied among landscapes, the total amount of habitat (10%), average quality of habitat, and locations of patch centroids remained constant in all landscapes.

Patch size disparity scenarios (low and high) were created by drawing individual patch sizes from 2 sets of bimodal distributions (with means of 125 size units) and modes of 100 and 150 in the low disparity scenario (hereafter, size disparity 50), and 50 and 200 for the high disparity scenario (hereafter, size disparity 150) and adjusted to meet total habitat amount criteria. In addition, we explored two scenarios in which the distribution of patch qualities varied to lesser and greater extents among patches. We assigned patches a habitat quality score (1–100 resource units), drawn from bimodal distributions (means = 50): to create a low disparity scenario (using modes of 40 and 60, hereafter referred to as quality disparity 20) and a high disparity scenario (using modes of 20 and 80, hereafter referred to as quality disparity 40) and adjusted to meet landscape quality criteria. We varied the proximity of high‐ to low‐quality patches using two patterns. Patches were either arranged along a habitat quality gradient, such that lowest and highest quality patches were clustered on opposite sides of the landscape (Fig. 1A), or high‐ and low‐quality patches were interspersed and proximate (Fig. 1B).

### Population model and ecological profiles

**Figure 2:**
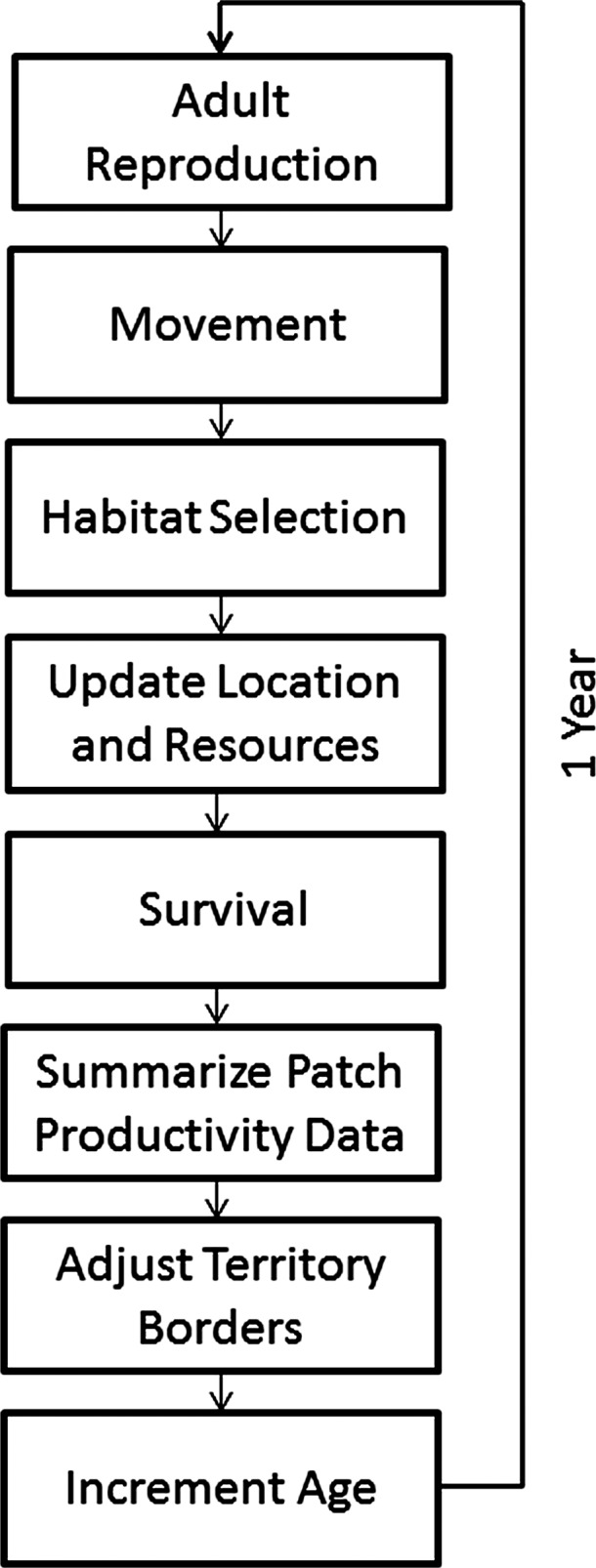
Annual cycle of simulation events as implemented in the naive population model. Initialization events, intermediary updating, and census functions are not shown.

We used the spatially explicit individual‐based modeling platform HexSim (version 2.3; Schumaker 2012), to construct a 3‐stage, females‐only population model (Fig. 2). Simulated animals interacted with habitat by moving through the landscape (200 × 231 hexagonal pixels) and searching for available territories. Each animal aimed to obtain an exclusive territory with the maximum possible resources within their maximum range size of 3 pixels (i.e., 3 pixels x quality score of 100 = 300 resource units). Individuals with smaller ranges were able to expand if adjacent areas were available for use in a later time step. Those able to meet the median resource requirement of 150 units by acquiring half of the maximum possible resources were given average survival rates of 0.74 for adults, and 0.52 for juveniles, based on mean survival rates and life history data for 155 avian populations (Stahl and Oli 2006) and 50 mammal populations (Heppell et al. 2000). Territory holders that acquired more or less than the median target level of habitat received linearly scaled survival rates that increased or decreased with their level of resources.

In initial simulations, we created a breath of ecological profiles to explore a range of responses to growth rates, environmental variation, and dispersal abilities. We were interested in examining the rate at which populations were able to recover from population downturns and return to carrying capacities, rather than exploring *r* vs. *K* life history strategies that often trade‐off survivorship and fecundity. Hence, we modeled populations with a range of growth rates. Population growth rates were the emergent properties of simulations, but were influenced by the ability of species to reproduce. We explored a range of growth rates by varying fecundity rates (0.75, 1.25, 1.75) among species. Territory‐holding adult females could reproduce, but floaters (without territories), juveniles, and subadults could not. Resulting growth (*λ*) values varied between ~0.5 and 2.0. We simulated three different levels of environmental variation that influenced the severity of periodic adverse conditions: no variation, minor variation, and major variation. Random selections from the lower half of a truncated normal distribution, that is, with a mean of 1.0 and standard deviations of 0.0 (no variation), 0.1 (minor variation), or 0.5 (major variation), determined the degree to which periodic environmental events reduced the survival rates for all individuals. The affected year was selected from a stratified random sample with 10 events/100 years. The affected year and associated severity were replicated across all simulation runs for a given scenario.

We created two movement scenarios for comparison by assigning juvenile (and new floater) dispersal distances that were sufficient to (1) reach the nearest few neighbor patches (200 pixels) or (2) move anywhere in the landscape (2000 pixels). In both these scenarios (nearest neighbors and unlimited neighbors), individuals moved in a quasirandom walk with forward momentum (75% autocorrelation in path direction). Exploration for a suitable territory was triggered if an individual encountered three adjacent hexagons of habitat after moving over 50 hexagons away from their natal territory, allowing individuals at the center of a patch to leave even the largest patches and generating a greater degree of passive dispersal away from smaller (rather than larger) patches. Dispersing individuals had a preference to move toward high‐quality habitat in their vicinity, with a strength that increased linearly with the pixel score. Hence, individuals were more likely to passively disperse away from low quality than high‐quality patches. Natal dispersal was mechanistically influenced by local density conditions. When density was high, natal dispersers travelled farther searching for a vacant location than if density was low.

#### Naive selection

To assess the influences of habitat and population factors on the strength of source–sink dynamics, we first evaluated the population outcomes using a naive habitat selection strategy. In this scenario, animals had limited knowledge of the landscape and were unaware of patch locations, habitat qualities, and densities beyond their individual experiences. Animals moved through the landscape in search of habitat, and those unable to obtain a suitable territory did not survive to the year. Individuals sought to occupy the highest quality, empty habitat within their search area, but had a limited spatial extent in which to make this determination after dispersing. Hence, individuals often occupied the first available, suitable territory that was encountered and remained there until death, with no subsequent movement to optimize fitness. The first animal to claim territory preempted subsequent individuals from using associated resources. In high‐density conditions, mechanistic emigration relocated juvenile dispersers to other patches.

For the naive habitat selection scenario, we simulated population dynamics using the above‐described modeling construct for 144 scenarios in a factorial design, combining 18 ecological profiles with eight neutral landscapes; however, rapid extinctions occurred in eight scenarios, reducing the sample size to 136. Simulations began with 100 females placed randomly within habitat patches, and population dynamics were assessed for the last 100 years of a 300‐year simulation, wherein population abundance generally fluctuated about a consistent mean. For each scenario, we ran 10 replicate simulations. Response surfaces were constructed to expand the parameter space and visualize the combinations of influential variables that yielded particularly strong or weak source–sink dynamics (i.e., top three factors). Additional levels of population growth potential (fecundity = 0.5, 1.25, 1.75) in combination with patch quality disparity (disparity alternative 40 with modes of 30 and 70), or environmental variation (0.25, 0.75, 1), were examined for visualization. Pattern, patch size disparity, and dispersal distance factors were held constant at levels that increased the strength of source–sink dynamics (See Appendix S1).

#### Informed selection

To evaluate the sensitivity of the drivers of source–sink strength to habitat awareness and selection, we evaluated an alternative scenario in which animals were cognizant of habitat quality and able to adjust their location through time to better optimize their fitness. Individuals did not have complete knowledge of their surroundings, but could discern low quality from high quality and could emigrate from lower‐quality patches in search of higher‐quality patches each year. Animals were also more discerning of habitat quality during movement and initial habitat selection (requiring high‐quality pixel values to trigger early stopping during movement), and their dispersal movements among patches were highly successful (individuals whose movement ended in the nonhabitat matrix were allowed to proceed to the nearest patch), emulating increased knowledge of the landscape. In lower‐quality patches, all individuals would emigrate each year in search of higher‐quality habitat. This quality‐induced emigration was required for all individuals in the lowest quality patch in each landscape and any other patches with mean pixel scores <31/100, implicating another two low‐quality patches in higher‐quality disparity scenarios (quality disparity 20, 60, and additionally 40; see below). Preemptive habitat selection was strengthened by allowing older individuals to preempt younger individuals arriving at the same location.

The informed habitat selection simulations focused on a subset of parameter space, chosen to maximize the breadth of parameter space for variables found to be important in the initial, naive scenario. Specifically, we evaluated the relative influences of the following drivers on the emergent strengths of sources and sinks using the following 36 factorial combinations: population growth potential (fecundity = 0.50, 1.00, 1.75), habitat quality disparity (alternatives 20 and 60; along with 40 which uses quality modes of 30 and 70), the pattern of high‐ and low‐quality patches (interspersed vs. gradient), and the degree of environmental variation (stochasticity SD = 0, 1.0). Based on the results of the naive selection strategy, we held patch size disparity and dispersal distance variables constant at their strongest levels.

### Source–sink metrics and analysis

We recorded the locations of individual births, immigrations, deaths, and emigrations, summarized the data for each patch, and assigned a productivity metric equal to births–deaths to each patch. Patches were deemed sources if local births exceeded deaths and sinks if deaths exceeded births over a 100‐year period. Movement among patches was accounted for in this metric by the individuals' subsequent contribution to the local birth or death tally, and using this construct, births–deaths approximates emigration – immigration at a steady state (Schumaker et al. 2014). To assess the system‐wide strength of source–sink dynamics, we also developed a second metric called productivity disparity, calculated as the difference between the average productivity (*b*–*d*) values of all sources and the average productivity values of all sinks in each landscape. At steady state, we expected a little variability in the source–sink status and the strength of patches in each scenario; therefore, demographic and movement information was averaged across the 10 replicate simulations. We assessed the influences of habitat and population factors on productivity disparity using analysis of variance (ANOVA) and ranked each factor using standardized effect sizes.

Alternative response metrics were also explored in the naive selection analysis to assess the sensitivity of the order of importance of the habitat and population traits to the choice of metrics. We examined metrics that used emigration and immigration data to weight patch productivity values and functionally describe patch contribution to the regional network. We also calculated the long‐term difference in productivity between the single most productive source and the single most consumptive sink in each landscape. Importance rankings were generally robust to the chosen metric, with the same top two factors being consistently identified across all models, and only minor variation in rankings observed among the less influential variables. We used the metric (productivity disparity) associated with the highest R^2^ model value to select the response variable in the naive selection models and retained the same metric for the informed selection models. For the naive selection scenario, we subsequently examined the relative influence of supporting factors while holding the most influential variable constant.

## Results

### Naive selection scenarios – general observations

**Figure 3:**
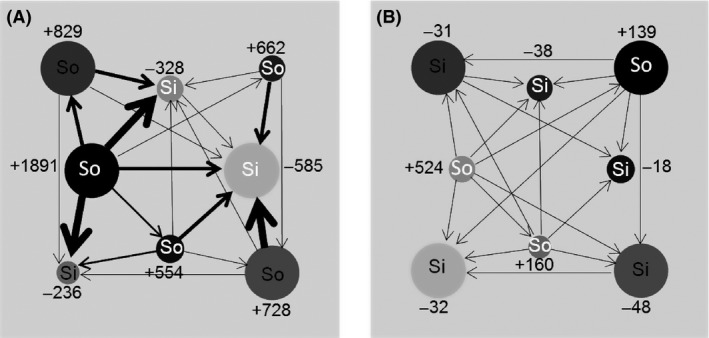
Study system yielding the strongest single source (A), and a contrasting system with weaker source‐sink dynamics and sources and sinks (B). The strength of sources (So), sinks (Si) are represented by their average annual local productivity (births‐deaths). Net movements among habitat patches are represented by arrows, with thicker lines indicating greater net annual movement, averaged among replicates. Exchanges of ≤1 net individuals were excluded. Darker circles represent higher‐quality habitat patches.

Despite each landscape containing an equal number of high‐ and low‐quality patches, the observed number of sources and sinks ranged from 7 sources and 1 sink to 2 sources and 6 sinks at stable state. We did not observe any systems with only sources or only sinks at equilibrium. The system with the highest productivity disparity had the following characteristics: highest growth (fecundity) rate and highest patch quality disparity levels, no environmental variation, high quality interspersed with low‐quality patches, and a limited dispersal ability (along with low patch size disparity). These variables and associated levels also predicted the system with the single strongest source, with the substitution of higher patch size disparity over low size disparity (Fig. 3 –left). The system producing the single strongest sink was almost identical to that producing the strongest source, differing only in that the strongest sink scenario did not limit dispersal to the nearby neighbor patches.

We expected the weakest source–sink systems to be produced by the species–landscape combinations with the opposite characteristics of the strongest source–sink systems. Rapid extinction precluded analysis of this specific scenario, but of those that reached steady state, the weakest source–sink dynamics were produced by systems with the lowest fecundity and patch quality disparity. The weakest source–sink systems were also characterized by an intermediate level of stochastic variation, interspersed pattern of high‐ and low‐quality patches, and the limited dispersal. We expected that longer dispersal distances would weaken the effect of source–sink pattern on the severity of dynamics, and we observed this effect in gradient but not in interspersed landscapes.

The source–sink status of individual patches was not always easily inferred from local habitat quality. Although source status tended to be more predictable in stronger source–sink systems (e.g., Fig. 3 – left), source–sink status was less related to quality in weaker systems (e.g., Fig. 3 – right with opposing growth rates, levels of variation, and landscape patterns, and equivalent quality, size, and dispersal characteristics to those on the left). Under weaker source–sink dynamics, some of the higher‐quality patches behaved as weak population sinks, whereas some of the moderate‐quality patches were the most productive sources in the landscape.

Some degree of compensation was observed among growth and secondary factors. Productivity disparity was particularly responsive to reductions in environmental variation or increases in quality disparity when population growth rates were low. In response surfaces, the influence of population growth was modulated by habitat quality disparity (e.g., Figure S1 – Appendix). The strength of source–sink dynamics under low‐quality disparity was nearly a third of that observed under high disparity conditions, with a threshold increase in productivity disparity under a moderate quality disparity scenario. Higher levels of quality disparity yielded more incremental increases in productivity disparity with the increasing levels of population growth (Figure S1 – Appendix).

### Naive selection scenarios – factorial analysis

**Figure 4:**
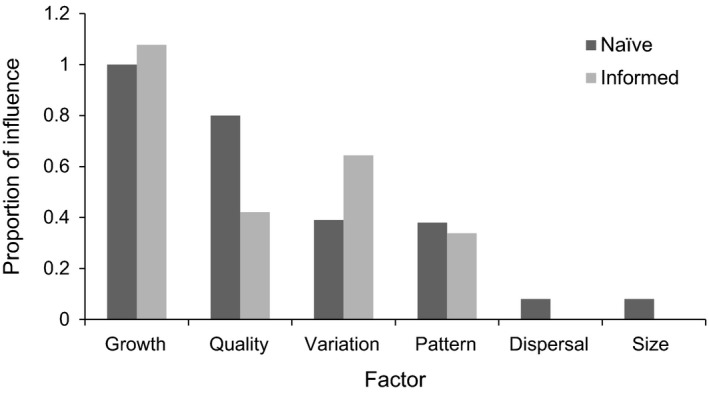
Proportional influences (Std *β*_i_/Std *β*_i__Max_) of key habitat and population factors in both naive and informed habitat selection scenarios.

When animals were limited in their ability to discern and respond to habitat quality in the naive habitat selection scenarios, population growth potential (as approximated with fecundity) had the greatest effect on productivity disparity (Table 1), with higher growth potential associated with the greater productivity differences among sources and sinks. The disparity in quality among habitat patches exerted the second greatest influence on productivity disparity, with greater quality disparity yielding stronger source–sink dynamics. Species responses to environmental variation as well as the proximity of sources to sinks (pattern) also ranked highly, accounting for ~39% and 38% of the influence of growth on productivity disparity (respectively, Fig. 4). All else being equal, lesser degrees of environmental variation and scenarios with high quality interspersed with low‐quality patches, rather than along a quality gradient, resulted in greater productivity disparity. Disparity in patch size and dispersal distances had weaker influences on the strength of source–sink dynamics, with 8% of the influence of the top ranked variable (Fig. 4). Shorter‐distance dispersal was associated with stronger source–sink dynamics, as was greater disparity in patch sizes. These variables were significant at *P *<* *0.1 and were modestly more influential when alternative metrics were tested.

**Table 1:** Influence of habitat and population variables on productivity disparity in the naive habitat selection scenario (*N* = 136), as described by the standardized effect sizes (*R* ^2^ = 0.86; *F* Ratio = 134.64; Prob > *F *= <0.0001)

Variable	Rank	Estimate	SE	*t* ratio	Prob>|*t*|	Std *β*
Intercept		67275.23	14598.50	4.61	<0.0001	0
Growth	1	60088.11	2772.75	21.67	<0.0001	0.7154
Quality	2	971.13	55.58	17.47	<0.0001	0.5754
Variation	3	−44426.36	5319.20	−8.35	<0.0001	−0.2759
Pattern [gr]	4	−2202.95	265.29	−8.30	<0.0001	−0.2713
Dispersal		−2.15	1.22	−1.76	0.0809	−0.0575
Size		−36.65	22.02	−1.66	0.0984	−0.0544

Growth = Population growth potential (fecundity); Variation = stochastic environmental variation; Pattern = proximity of high‐ to low‐quality patches; Quality = patch quality disparity; Size = patch size disparity; Dispersal = dispersal ability.

**Figure 5:**
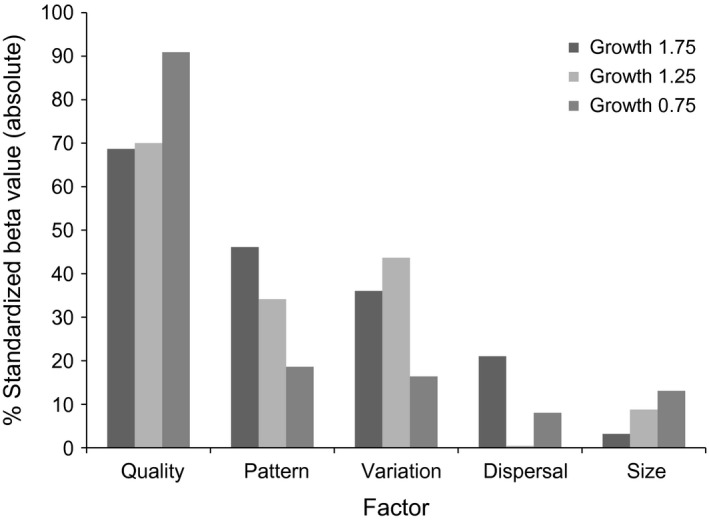
Relative influences (Absolute Std *β*_i_ values × 100) of key habitat and population factors, holding population growth levels constant at 1.75, 1.25, and 0.75 in naive habitat selection scenarios.

When population growth rate was held constant at each level, habitat quality consistently became the most important driver of source–sink strength (Fig. 5). The slowest growing populations displayed the greatest response to habitat quality disparity among patches, with lesser contributions from other variables. In contrast, the fastest growing populations displayed a diminished response to habitat quality although it was still the most influential factor.

### Informed selection scenarios – factorial analysis

When animals had increased knowledge of the landscape and were more successful at moving to and selecting better habitat, population growth potential still emerged as the most influential variable (Table 2) driving the strength of source–sink dynamics, with slightly greater influence than in the naive selection scenario. The rank order of the second‐most influential variable was altered compared to the naive scenario, with the influence of environmental variation becoming more important than patch quality disparity (Fig. 4).

**Table 2:** Influence of habitat and population variables on productivity disparity in the informed habitat selection scenario (*N* = 28), as described by the standardized effect sizes (*R* ^2^ = 0.78; *F* Ratio = 20.03; Prob > *F* = <0.0001)

Variable	Rank	Estimate	SE	*t* Ratio	Prob>|*t*|	Std *β*
Intercept		−21680.37	12406.52	−1.75	0.0939	0
Growth	1	43781.14	5878.86	7.45	<.0001	0.7707
Variation	2	−26373.48	5757.39	−4.58	0.0001	−0.4607
Quality	3	562.19	190.92	2.94	0.0073	0.3010
Pattern [gr]	4	−6873.41	2825.47	−2.43	0.0232	−0.2420

Growth, population growth potential; variation, stochastic environmental variation; pattern, proximity of high‐ to low‐quality patches; quality, patch quality disparity. Dispersal distance (short) and patch size disparity (level 150) were held constant among simulation scenarios.

The strongest mean difference among source and sink strength was observed in a scenario with the highest examined growth potential level (fecundity = 1.75), no environmental catastrophes (variation = 0.0), the greatest level of quality disparity among patches (80), and an interspersed pattern of high‐ and low‐quality patches. The habitat population scenario with the opposing characteristics that was expected to have the weakest source–sink dynamics went extinct before data were collected. The weakest observed dynamics were generated from the combined influences of the lowest growth level (fecundity = 0.5), higher environmental variability (1.0), a moderate level of patch quality disparity (40), and patches arranged along a gradient of qualities.

In general, the emergent source–sink systems that were influenced by informed habitat selection were weaker than those produced by naive habitat selection. On average, the informed habitat selection scenario produced source–sink dynamics that were 73% of the strength of those of the naive scenarios (using only comparable scenarios with short dispersal distances and high patch size disparity), with less variation in source–sink strength among populations and landscapes.

## Discussion

### Relative influence

Many factors have been found to incite source–sink dynamics, yet their relative contributions in strengthening dynamics remained relatively unexplored. In an effort to help identify the characteristics of systems that are likely to exhibit strong source–sink dynamics, we compared the roles of influential factors in strengthening source–sink dynamics in a series of controlled simulation experiments to generate hypotheses for future exploration with empirical data. Our results suggest that systems with species capable of rapid growth, occupying habitat patches with more disparate qualities among patches, and in relatively stable environments (i.e., fewer negative perturbations), are more likely to exhibit strong source–sink dynamics. The pattern of high‐ and low‐quality habitats was also influential in inciting and strengthening dynamics. Dispersal ability and differences in patch sizes had weaker impacts. Although some factors were much stronger drivers than others, strong source–sink dynamics emerged under a range of factors in multiple different scenarios, suggesting that multiple lines of data and inference are likely to be needed to predict the strength of source–sink dynamics.

In both the naive and informed habitat selection scenarios, the strongest driver of source–sink dynamics was population growth (i.e., fecundity). Although the intuitive expectation is that differential habitat quality drives source–sink dynamics, our simulations suggest that heterogeneous habitat quality provides an influential stage upon which density‐regulated populations can act to further strengthen source–sink dynamics. Smaller, slow‐growing populations operate more frequently below carrying capacity, where there is less impetus for density‐dependent emigration from natal patches and less exposure to survival and reproductive limitations. This weakened sources and sinks over populations are capable of faster slower growth in our simulations. Although stronger source–sink dynamics were associated with higher population sizes, this relationship was increasingly variable in regional populations with stronger dynamics, indicating that that population size alone is unlikely to generally predict source–sink severity. The combination of population growth potential and population size relative to carrying capacities may better predict the strength of source–sink dynamics. As demographic rates are themselves a function of inherent species characteristics, as well as current and past environmental drivers (Treurnicht et al. 2016), future research could decompose population growth in specific systems to gain a more complete indication of the key demographic drivers influencing source–sink dynamics.

Our results indicate that systems with greater disparity in habitat quality among patches are likely to incite and produce stronger source–sink dynamics, with the strongest dynamics emerging in systems with both high quality disparity and population growth. Species with inherently lower capacities for population growth (e.g., lower fecundity rates) with a naive habitat selection strategy may be particularly sensitive to the disparity of habitat quality among patches (e.g., growth 0.75 in Fig. 5), and the ensuing source–sink dynamics may be largely driven by habitat quality differences. Species subject to greater intensities and durations of density‐dependant population regulation (e.g., growth = 1.75 in Fig. 5) were still primarily influenced by habitat quality disparity, but variation, pattern, and dispersal factors weighted in more heavily than in low‐growth scenarios. Across different kinds of systems, habitat quality information is likely to be helpful in inferring the presence of source–sink dynamics; however, this information alone is unlikely to accurately predict the strength of dynamics. Habitat quality disparity may better indicate the strength of source–sink dynamics among populations of the same species or groups of species with a similar low potential for population growth. Response surfaces indicate that threshold effects may exist (e.g., low quality = ~40% of the value of high quality), above which quality disparity among patches may produce much stronger source–sink dynamics. Such thresholds may complicate source–sink strength inferences based on quality disparity data. Habitat quality disparity may also be more important than described here for species who are unable to expand their territories to compensate for a low quality or quantity of resources, or philopatric social organisms that share resources (e.g., scramble competition) with an unwillingness to relocate or break up the group.

Environmental stability and the spatial pattern of sources and sinks were also important in predicting the strength of sources–sink dynamics. When used in combination with population growth and quality disparity information, environmental stability may indicate the presence and strength of source–sink dynamics, particularly for species with the informed habitat selection. Temporal fluctuations in demographic rates can influence the magnitude and duration of density‐dependent effects (e.g., alteration of dispersal patterns; Holt 1996; Virgl and Messier 2000) and influence the strength of source–sink dynamics. In our model, populations subject to weak environmental variation experienced only minor reductions in abundance and maintained higher average population sizes than populations more strongly affected by variation. Through time, higher‐quality patches were strengthened as a result of more consistent occupancy and higher local population densities. Emigration from highly occupied patches to lesser quality patches strengthened sinks and created greater disparity in productivity among sources and sinks. For species with the informed habitat selection, population stability leading to density‐dependent movement was more important than habitat quality disparity. With the recognition of lower‐quality habitat, the ability to leave in search of better habitat with higher dispersal success, informed species could seek out locations that better optimize their fitness, predictably weakening the impact of habitat quality disparity. As our examination was restricted to periodic stressors or catastrophes, future studies could invoke different kinds of environmental stochasticity affecting population dynamics including positive autocorrelation (e.g., Crone 2016) to further assess the sensitivity of source‐sink strength to environmental variability.

The likelihood that individuals leaving sources can successfully reach a sink is generally expected to affect the strength of sources and sinks (Pulliam 1988; Walters 2001; Holland et al. 2009). Sinks that are proximate to sources (e.g., interspersed) are more likely to be encountered and occupied by emigrants from sources, strengthening nearby sinks and their contribution to overall dynamics. Conversely, clustered sinks (e.g., low‐quality patches along a habitat quality gradient) can result in lower population persistence (Matthews and Gonzalez 2007), indicating that lower population sizes and high local extinction rates may reduce the long‐term severity of sinks and diminish the overall strength of source–sink dynamics. The pattern of high‐ and low‐quality patches modified the strength of source–sink dynamics in all scenarios, with interspersed quality patches strengthening, and gradients weakening source–sink dynamics similarly in both naive and informed selection scenarios. We found limited support for dispersal ability mediating the effect of spatial patterning of habitat quality on the strength of source–sink dynamics. In gradient landscapes, naive species with unlimited dispersal had weaker source–sink dynamics compared to dispersal‐limited species. However, in interspersed landscapes, source–sink severity was similar among short and long dispersers. Similarly, Walters (2007) found that the effects of breeding patch configuration outweighed dispersal characteristics (including distance) on dispersal success, suggesting that the influence of source–sink patterning may generally outweigh that of dispersal in strengthening sources and sinks.

The exchange of individuals among populations is a necessary condition for source–sink dynamics to arise, as the existence of sink populations often relies on immigration from sources (Gunderson et al. 2001; Schooley and Branch 2007). Hence, the increased ability of animals to travel across the landscape in search of optimal habitat (relative to interpatch distances) was expected and observed to weaken source–sink dynamics. In the naive habitat selection scenario, animals with short dispersal distances (relative to interpatch distances) were limited to settling in nearby patches regardless of their quality, strengthening proximate sinks and source–sink dynamics. Animals capable of longer distance dispersal (but similarly the limited perceptual ranges) were able to migrate to distant, more isolated patches, increasing their immigration and occupancy rates, and weakening the effect of source–sink pattern on the severity of dynamics. In our limited exploration of dispersal, an animal's ability to search the landscape for new habitat was not a key factor driving the strength of source–sink dynamics. However, dispersal ability might be more important in species that rely more on random explorative searches for habitat, with long dispersal abilities coupled with higher habitat selection criteria than those examined here, and in landscapes with a complex, heterogeneous matrix.

All else being equal, we expected that landscapes with disparate patch sizes would have stronger sources than those with similar patch sizes. Larger patches have greater capacities, receive more immigrants via diffusion movements in the matrix (i.e., higher encounter rates; (Bowman et al. 2002), and emit fewer emigrants resulting from passive dispersal, which can strengthen source patches (Walters 2001). In turn, stronger sources would be expected to create stronger sinks and source–sink dynamics by increasing sink immigration and occupancy rates. Although more disparate patch sizes generally strengthened source–sink dynamics*,* patch size disparity was not a prominent driver of source–sink strength, owing in part to the effects of other factors on emigration routes and rates. Patch size disparities may be more important in species that rely more on passive dispersal and random diffusion than explored here, and in systems with strong responses to patch edges.

### Implications for source–sink populations

Hypothetical source–sink systems provide a controlled means of gauging the relative influences of a number of general ecological conditions and can serve as a tool for generating testable hypotheses. These models lack the context‐specific details of complex natural systems, yet in an informal evaluation of modeled source–sink dynamics for Black‐capped vireos and Ord's kangaroo rats based on Heinrichs et al. (2015), we found support for population growth rates indicating the strength of source–sink dynamics across systems. Heinrichs et al. incorporated species' life history details (demography, movement, density‐dependent habitat selection) and landscape information into realistic spatially explicit individual‐based models and then computed habitat patch productivity as has been carried out here. These population growth rates successfully predicted the rank order of the overall strength of source–sink dynamics across these case study systems and scenarios. Despite this, the degree to which the strength of source–sink dynamics can be predicted in individual systems may still depend on the importance of case‐specific details and dependencies (e.g., Loehle 2012); hence, empirical data are required to further test and develop these hypotheses.

Results from theoretical source–sink research are often criticized for being difficult to operationalize in empirical systems, and management based on demographic and source–sink concepts is often constrained by practical constraints (e.g., Kerr et al. 2016), including the costs of intensive data collection. Yet, uncertainty in demographic conditions, source–sink characterizations, and the strength of source–sink systems can undermine the management efforts (Barthold et al. 2016; Griffith et al. 2016). A conceptual understanding of the nature of dynamics among subpopulations could be helpful in guiding and targeting the intensive resources required to collect data to assess and evaluate cross‐system patterns of source–sink intensities. Well‐developed and tested theory predicting the expected strength of source–sink systems can provide a low effort screening tool to identify situations in which source–sink analyses should be undertaken and used to inform management strategies. Conservation and management actions may need to be different for systems with weak versus strong source–sink dynamics, and approaches and decisions made for systems with weak dynamics may not hold for those with strong dynamics. Knowledge of the strength of source–sink dynamics present within a system should be also helpful in indicating the degree of interdependency and the importance of connectivity among populations, and in identifying actions that could be used to alter the severity of source–sink dynamics, particularly for declining species. For instance, systems with particularly strong sources and/or sinks may have patches that disproportionately contribute to and drive regional population dynamics (Schlaepfer et al. 2002; Kawecki 2004; Runge et al. 2006). In such metapopulations, it may be particularly important to accurately identify and assess the strengths and contributions of sources and sinks prior to the selection of local habitats for protection, restoration, or monitoring population trajectories. For example, the strongest and most central source (*b*–*d* = 1891) in Figure 3A drives the performance of all of its neighbors and would likely be a primary target for preservation. Weaker sources or sinks may be targets for habitat restoration, and strong sinks (e.g., *b*–*d* = −585 in Fig. 3A) may also be particularly suitable sites for monitoring changes in source reproductive output (Jonzen et al. 2005) or targeted for habitat removal.

Lastly, generalizations about the factors influencing source–sink severity could be helpful in identifying systems wherein source–sink dynamics may be difficult to detect and where local source–sink identifications might prove difficult or require increased accuracy. Lesser differences in productivity among sources and sinks are expected in systems with weaker source–sink dynamics, making the status of local populations more difficult to identify with confidence, particularly given the difficulty in collecting demographic information and the uncertainty inherent in such data (Runge et al. 2006; Johnson 2007; Robinson and Hoover 2011). Our results suggest that demographic differences among subpopulations may be easier to detect and measure in populations that are not continually challenged by stochastic events, capable of rapid growth, and that inhabit heterogeneous quality landscapes with interspersed high‐ and low‐quality patches. Conversely, it should be more difficult to detect and measure source–sink dynamics in slow‐growing populations, highly variable environments, and where a subtle gradient of habitat quality exists. This suggests that if all else is equal, populations wherein reproduction is consistently (intrinsically or extrinsically) suppressed, and populations subject to significant periodic survival stressors (e.g., weather events, exposure to toxins, disease, interspecific interactions), are less likely to exhibit large differences in demographic measurements. Similarly, populations inhabiting landscapes with gradations in habitat quality (e.g., mirroring the transition of underlying vegetation or geologic conditions) are expected to have weaker sources and sinks. In weak source–sink systems, data collection may need to be more comprehensive to detect differences in productivity among local populations and determine their meaning and relevancy for habitat and population management.

## Data accessibility

Data for this paper (model inputs, scenario files, HexSim spatial data, etc.) are publically accessible in an archive database (Dryad). The software and user's guide can be downloaded from http://www.hexsim.net.

## Conflict of Interest

None declared.

## Supporting information


**Appendix S1**. Supplementary Data (Naive Scenario).
**Table S1**. Influence of habitat and population variables on productivity disparity holding the population growth level constant at 1.75 in the naive scenario (*N* = 48), as described by standardized effect sizes (*R* ^2^ = 0.86; *F* Ratio = 51.35; Prob > *F* = <0.0001).
**Table S2**. Influence of habitat and population variables on productivity disparity holding the population growth level constant at 1.25 in the naive scenario (*N* = 48), as described by standardized effect sizes (*R* ^2^ = 0.81; *F* Ratio = 34.78; Prob > *F* = <0.0001).
**Table S3**. Influence of habitat and population variables on productivity disparity holding the population growth level constant at 0.75 in the naive scenario (*N* = 40), as described by standardized effect sizes (*R* ^2^ = 0.79; *F* Ratio = 26.14; Prob > *F* = <0.0001).
**Figure S1**. Productivity disparity response surfaces for the top ranked factor, growth, with (A) quality disparity (rank = 2), and (B) environmental variation (rank = 3) in the naive scenario.
