# Supplementary material for: Intrinsic and extrinsic drivers of source–sink dynamics
Source: Ecol Evol. 2016 Feb 22;6(4):892–904. doi: 10.1002/ece3.2029 (PMC4761763; doi:10.1002/ece3.2029)
Supplement: Supplementary file 1 — Appendix S1. Supplementary Data (Naive Scenario). Table S1. Influence of habitat and population variables on productivity disparity holding the population growth level constant at 1.75 in the naive scenario (N = 48), as described by standardized effect sizes (R 2 = 0.86; F Ratio = 51.35; Prob > F = <0.0001). Table S2. Influence of habitat and population variables on productivity disparity holding the population growth level constant at 1.25 in the naive scenario (N = 48), as described by standardized effect sizes (R 2 = 0.81; F Ratio = 34.78; Prob > F = <0.0001). Table S3. Influence of habitat and population variables on productivity disparity holding the population growth level constant at 0.75 in the naive scenario (N = 40), as described by standardized effect sizes (R 2 = 0.79; F Ratio = 26.14; Prob > F = <0.0001). Figure S1. Productivity disparity response surfaces for the top ranked factor, growth, with (A) quality disparity (rank = 2), and (B) environmental variation (rank = 3) in the naive scenario. [file ECE3-6-0892-s001.docx]

Appendix S1: Supplementary Data (Naive Scenario)

*Secondary Factor Analysis – Constant Population Growth*

Table S1: Influence of habitat and population variables on productivity disparity holding the population growth level constant at 1.75 in the naive scenario (N =48), as described by standardized effect sizes (R^2^ = 0.86; F Ratio = 51.35; Prob >F = < 0.0001).

| Variable | Rank | Estimate | SE | t Ratio | Prob>\|t\| | Std Beta |
| --- | --- | --- | --- | --- | --- | --- |
| Intercept |  | 60249.54 | 6111.37 | 9.86 | <.0001* | 0 |
| Quality | 1 | 1076.46 | 90.70 | 11.87 | <.0001* | 0.6867 |
| Pattern [gr] | 2 | -14456.28 | 1813..92 | -7.97 | <.0001* | -0.4611 |
| Variation | 3 | -52345.10 | 8396.84 | -6.23 | <.0001* | -0.3607 |
| Dispersal | 4 | -7.32 | 2.02 | -3.63 | 0.0008* | -0.2102 |
| Size |  | -19.91 | 36.28 | -0.55 | 0.5861 | -0.0318 |

Variation = stochastic environmental variation; Pattern = proximity of high to low quality patches; Quality = patch quality disparity; Size = patch size disparity; Dispersal = dispersal ability.

Table S2: Influence of habitat and population variables on productivity disparity holding the population growth level constant at 1.25 in the naive scenario (N =48), as described by standardized effect sizes (R^2^ = 0.81; F Ratio = 34.78; Prob >F = < 0.0001).

| Variable | Rank | Estimate | SE | t Ratio | Prob>\|t\| | Std Beta |
| --- | --- | --- | --- | --- | --- | --- |
| Intercept |  | 32854.59 | 5780.95 | 5.68 | <.0001* | 0 |
| Quality | 1 | 882.74 | 85.79 | 10.29 | <.0001* | 0.7003 |
| Variation | 2 | -50958.56 | 7942.90 | -6.42 | <.0001* | -0.4367 |
| Pattern [gr] | 3 | -8611.50 | 1715.86 | -5.02 | <.0001* | -0.3416 |
| Size |  | -44.17 | 34.32 | -1.29 | 0.2051 | -0.0876 |
| Dispersal |  | -0.13 | 1.91 | -0.07 | 0.9473 | -0.0045 |

Variation = stochastic environmental variation; Pattern = proximity of high to low quality patches; Quality = patch quality disparity; Size = patch size disparity; Dispersal = dispersal ability.

Table S3: Influence of habitat and population variables on productivity disparity holding the population growth level constant at 0.75 in the naive scenario (N =40), as described by standardized effect sizes (R^2^ = 0.79; F Ratio = 26.14; Prob >F = < 0.0001)

| Variable | Rank | Estimate | SE | t Ratio | Prob>\|t\| | Std Beta |
| --- | --- | --- | --- | --- | --- | --- |
| Intercept |  | -2797.26 | 4834.00 | -058 | 0.5666 | 0 |
| Quality | 1 | 846.76 | 79.03 | 10.71 | <.0001* | 0.9092 |
| Pattern [gr] | 2 | -3402.34 | 1421.95 | -2.39 | 0.0224* | -0.1864 |
| Variation |  | -16160.19 | 8349.95 | -1.94 | 0.0613 | -0.1642 |
| Size |  | -47.73 | 28.44 | -1.68 | 0.1024 | -0.1308 |
| Dispersal |  | -3402.34 | 1.58 | 1.03 | 0.3114 | 0.0801 |

Variation = stochastic environmental variation; Pattern = proximity of high to low quality patches; Quality = patch quality disparity; Size = patch size disparity; Dispersal = dispersal ability.

*Response Surfaces*

Response surfaces (Figure A1) were constructed to expand the parameter space and visualize combinations of influential variables that yielded particularly strong or weak source-sink dynamics (i.e., top three factors). Lesser-ranked factors held constant at levels that produced the maximum influence (i.e., levels that generally increased the strength of source-sink dynamics). Pattern was held constant with high quality patches interspersed with low quality patches, as was patch size disparity (using highly disparate patch sizes with disparity level 150), and dispersal was limited to the nearest few neighboring patches (200 pixels). In scenarios examining the relationship between fecundity and quality disparity, there was no environmental variation (i.e., static). In fecundity-variation scenarios, quality disparity was held constant with disparity level 60 (modes of 20 and 80).


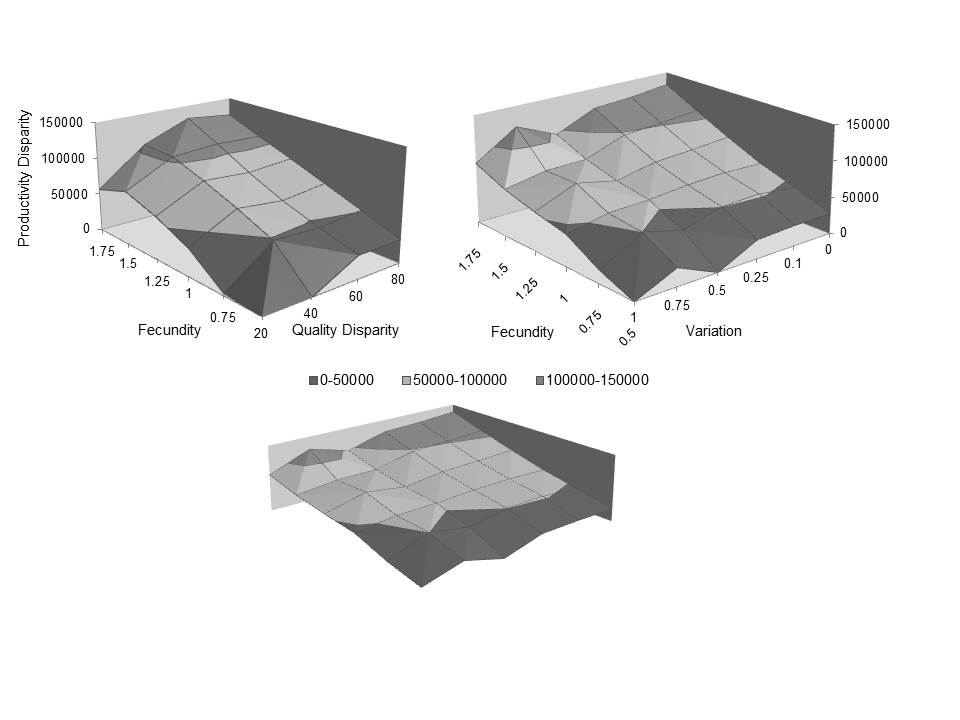


Figure S1. Productivity disparity response surfaces for the top ranked factor, growth, with a) quality disparity (rank = 2), and b) environmental variation (rank = 3) in the naive scenario.
